# When and how scientists influence technological performance: A moderated mediation model

**DOI:** 10.1371/journal.pone.0297022

**Published:** 2024-01-25

**Authors:** Jinxing Ji, Jieyu Song, Na Liu

**Affiliations:** 1 School of Management, Zhongnan University of Economics and Law, Wuhan, China; 2 School of Management, Shandong Technology and Business University, Yantai, China; University of Greenwich, UNITED KINGDOM

## Abstract

Previous studies have primarily investigated scientists’ direct impact on technological performance. Expanding on this, the study explores the nuanced ways and timing through which scientists influence team-level technological performance. By integrating knowledge-based and network dynamics theories, the study establishes and assesses membership turnover as a significant mediator of the science–technological performance process. Furthermore, it investigates the moderating effects of team internationalization and coreness on the mediation effects. Employing an unbalanced panel dataset from Huawei and Intel from 2000 to 2022, the study applied the Tobit and Negative Binomial models and conducted robustness tests for data analysis. The findings support the indirect influence of scientists within an invention team on the quantity and quality of inventions through membership turnover. Moreover, team internationalization diminishes the relationship between membership turnover and the quantity and quality of inventions, thereby impairing scientists’ indirect effects on technological performance through membership turnover. Team coreness enhances the relationship between membership turnover and the quantity and quality of inventions, strengthening the indirect impact of scientists on these dimensions through membership turnover.

## Introduction

Invention teams serve as the primary corporate strategy and operational units of technological innovations [1, 2]. Members are more connected among themselves than among outsiders. Thus, firm-based invention teams are characterized by tight connections and high cohesion. Team technological performance refers to the quantity and quality of inventions team members create through frequent collaboration [3–5].

According to knowledge-based theory, the essence of innovation embraces knowledge creation and application [6]. Previous studies have addressed the relevance of scientific knowledge in technological development and diffusion [7, 8]. Scientists can enhance the knowledge search process and facilitate access to distant knowledge [9], because of their contribution to invaluable scientific expertise. Consequently, teams with more scientists can access knowledge across various technological domains, leading to more innovative solutions [10]. However, scientific knowledge is often implicit and sticky, incurring costs for transferring and integrating scientific and technological knowledge [2, 11], and negatively impacting technological performance. These inconsistent results indicate that communication and dissemination of scientific knowledge from scientists outside the team to the invention team is difficult. To optimize technological advancements, we expect an invention team to internalize some scientific knowledge by incorporating scientists into the team. Our research aims to address a crucial gap in research on the influence and internal mechanisms of incorporating scientists into a team on technological performance. We attempt to understand whether and how leveraging scientists within an invention team allows the team to improve the quantity and quality of inventions.

Based on innovation network theory, collaboration networks facilitate innovation outcomes. A scholarly consensus supports the positive impact of network structures such as core/periphery and small-world networks on technological performance [12, 13]. Network dynamics theory posits that collaboration innovation is a dynamic process, inventors must continually seek out novel knowledge and collaborative opportunities through forming and dissolving of partnerships [14, 15]. In today’s fast-paced business environment, network dynamics are integral in technological advancement and essential for invention teams aiming to maintain a competitive edge. While existing studies have focused on the impact of network dynamics on technological innovation performance, they have overlooked the formation mechanisms triggering these network dynamics.

According to previous research, micro-foundations such as agency behavior, opportunity, and habits drive the changing structure of collaboration networks [16]. Scientists publish scientific papers and file patents in an invention team, while pure inventors only apply for patents [17]. The distinction in research paradigms, work habits, and knowledge base potentially influences the persistence of team collaboration [18], a critical factor influencing the success of an invention team. Thus, by implementing membership turnover as a mediator in the theoretical model, we better understand of how scientists within an invention team contribute to technological performance through membership turnover.

Furthermore, we expanded our mediation model by accounting for contingencies in the direct effect of membership turnover on technological performance. Given the unique characteristics of each invention team, it is crucial to explore how the influence of membership turnover on technological performance is affected by specific team traits. The characteristics of team internationalization and team coreness reflect the team’s capacity to control and search for diverse resources [19, 20]. This study explores the moderating effects of team internationalization and coreness on the relationship between membership turnover and technological performance. Specifically, it assesses how the levels of team internationalization and coreness affect the association between membership turnover and technological performance.

The study integrated knowledge-based and network dynamics theories to propose a moderated mediation model and formulate our hypotheses. Hypotheses 1a and 1b relate to scientists’ impact on two dimensions of technological performance, while Hypotheses 2a and 2b concern the impact of membership turnover on technological performance. Addressing the mediating role of membership turnover in these relationships, Hypotheses 3a and 3b were developed. Hypotheses 4a, 4b, 5a, and 5b explore the moderating effects of team internationalization and team coreness on the relationship between membership turnover and technological performance. Finally, Hypotheses 6a, 6b, 6c, and 6d discuss the moderating effect of these two moderators on the indirect relationships between scientists and technological performance.

This study makes several contributions to existing literature. First, it contributes to the literature on technological innovation by evaluating scientists within an invention team as critical predictors of team-level performance. It proposes a moderated mediation model that explains and empirically tests how two types of technological performance (invention quantity and quality) vary in invention teams with scientists, complementing the neglected detailed links between scientists within the team and technological performance. Second, the study significantly contributes to the literature on network dynamics by identifying membership turnover as a distinct mediation mechanism through which scientists within an invention team indirectly influence technological performance. Third, it expands the existing literature on the relationship between scientists and technological performance by investigating the moderating impact of team internationalization and team coreness on the mediating effect of membership turnover. This study provides insights into the conditions under which scientists impact a team’s technological performance, thereby clarifying the boundary conditions for the indirect effect of scientists on technological performance.

The following section outlines the conceptual background and hypotheses. Subsequently, it presents the study’s data, methodology, and empirical findings, drawing from panel data spanning a substantial period (2000–2022). This paper concludes with a discussion and suggestions for future research.

## Theoretical background and hypothesis

### Scientists and technological performance

Knowledge-based theory suggests that improving technological performance is intricately linked to boundary-spanning mechanisms, enabling the application of scientific knowledge to technological inventions [7, 9, 21]. In an invention team, scientists encompass members engaged in scientific and technological innovation, with innovation outcomes manifesting in scientific publications and patents. Conversely, pure inventors exclusively contribute to technological innovation and patent applications. Scientists within an invention team bridge science and technology [17, 22], reflecting the team’s ability to absorb and transform scientific knowledge, thereby influencing the quantity and quality of technological inventions.

The study posits that scientists within an invention team, with strong backgrounds in scientific and technological knowledge [23], positively impact a team’s technological performance through innovation strategy formulation, knowledge search, and integration. In an invention team, scientists facilitate the rapid identification of industrial technology development directions, allowing for the formulation of exploratory and commercial technological innovation strategies [24]. Their profound comprehension of scientific principles underlying technological phenomena enables the discovery of new technological trends, fostering innovation and advancement.

Additionally, given scientists’ affiliation with the invention team, they possess familiarity with the technology market, enabling easy access to information on market demand and technological changes. Serving as connectors translating scientific knowledge into applied technology, these scientists in the team emphasize the exploratory aspect of technological inventions and express concerns about the prospects of technology commercialization [25].

Scientists optimize the invention team’s scientific and technological knowledge search process, minimizing associated costs [9]. Their social capital, representing the accumulated scientific and technological resources through social ties, facilitates the effective transfer of knowledge and reduces search costs [26]. For example, social capital in science fosters persistent collaborations with universities or research institutes at a lower cost [15, 27], whereas technological social capital strengthens ties with technological alliances [4, 28]. Scientists also contribute to mitigating team uncertainty during knowledge searches. Scientists within a team leverage their inventive experience and scientific knowledge to evaluate and recognize valuable scientific knowledge efficiently, enhancing the team’s technological search.

Scientists provide advantages for absorbing and integrating of scientific and technological knowledge [2]. In contrast, an invention team lacking this ability may face increased learning costs with a surplus of scientific and technological knowledge, as applying scientific knowledge to technological inventions can be challenging. A team of scientists forms a technological-scientific community capable of generating feasible ideas for translating scientific knowledge into applied technology through mutual knowledge exchange [27]. Hence, scientists positively impact the team’s absorptive capacity and knowledge integration.

However, scientists may yield diminishing returns for teams when the costs, arising from an excessive number of scientists within an invention team, surpass the benefits. Balancing scientific and applied research may be essential for fostering technological performance [29]. Scientists within a team may create significant tension with pure inventors. The distinctions between scientists and pure innovators, including knowledge scope and innovation standards, persist owing to their focus on separate domains [10]. Scientific innovation will likely affect breakthrough inventions, advancing new technological trajectories [30]. Conversely, pure inventors lacking scientific experience may exhibit higher inventive inertia, decreased motivation to seek new knowledge beyond their expertise, and a tendency toward exploitative innovation. Therefore, an excessive number of scientists within an invention team may disrupt the balance with pure inventors, exacerbating gaps and contradictions regarding the knowledge scope and innovation standards between the two groups.

Owing to the high uncertainty of scientific research [31], invention teams struggle to be rewarded in the short term, contrary to their goal of maximizing interests. Therefore, conducting extensive scientific studies becomes challenging for these teams. Moreover, Integrating scientific and technological knowledge is complex [2, 24]. Being implicit and sticky, scientific knowledge poses challenges in recombining with technological knowledge elements [11]. While an excessive number of scientists results in overproducing sticky knowledge, it simultaneously narrows the effective integration of scientific and technological knowledge. Therefore, an excessive number of scientists within a team can impose greater obstacles to the effective integration of scientific and technological knowledge spanning broader scopes.

It is crucial to expect significant benefits from an invention team with a moderate presence of scientists. This moderation enhances the integration of scientific and technological knowledge and minimizes the costs and risks associated with excessive scientists, thereby maximizing technological performance. Therefore, we propose the following hypotheses:

H1a: Scientists within an invention team have an inverted U-shaped effect on the team’s invention quantity.

H1b: Scientists within an invention team have an inverted U-shaped effect on the team’s invention quality.

### Membership turnover and technological performance

**Fig 1:**
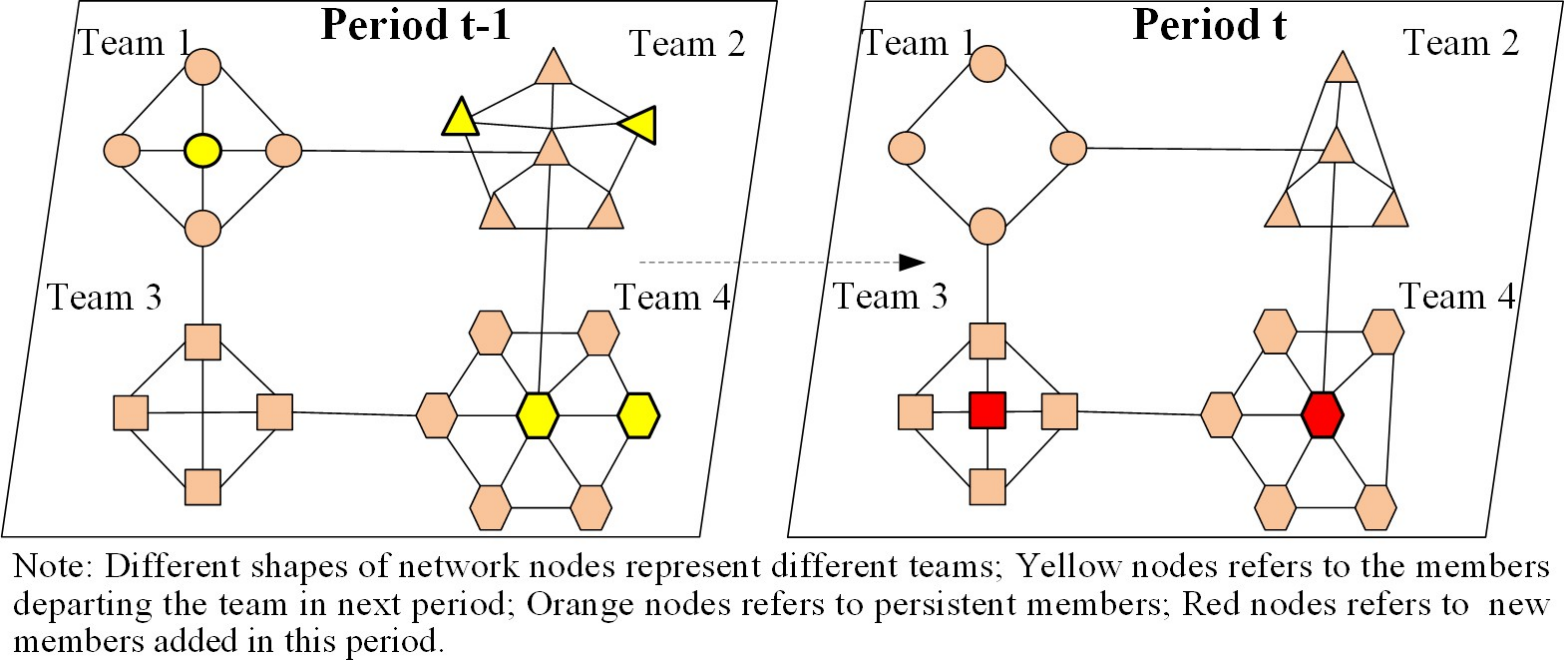
Dynamics of members within invention teams.

This study, identifies invention teams as the dense and nonoverlapping structural groups of inventors within a co-inventor network. In any team, members are more connected with each other than those outside the team. Network dynamics theory suggests that network architecture can change, by adding or subtracting network nodes and creating or dissolving ties between them [16]. We adopt the approach of Sytch and Tatarynowicz (2014), utilizing a modification of network nodes to indicate a team’s membership turnover, reflecting the arrival of new members and the departure of existing ones [32]. Fig 1 depicts the membership turnover in the four invention teams from period t-1 to t.

The association between network dynamics and technological innovation has recently become the focus of several studies. Kumar and Zaheer (2019) proposed that network stability may negatively influence innovation performance, while network dynamics are positively associated with innovation performance [28]. First, an invention team with moderate membership turnover can offer diverse resources [15, 32]. This turnover, involving the departure of old members and the introduction of new ones, creates opportunities for new partnerships and access to resource pools in other teams. Second, a moderate membership turnover can reduce conformity pressures and avoid homogenizing tendencies [16, 32]. Invention teams are cohesive groups of inventors, and individual behaviors are easily influenced by other members. When the individuals’ inventive behaviors conflict with the team’s overall innovation strategies, they are suppressed because of the opposition of other team members. The arrival of new members can expose team members to novel ideas and collaborative strategies, which can help enhance the team’s technological performance.

However, membership turnovers within an invention team may eventually result in reduced technological performance. First, a high rate of membership turnover may threaten the stability of collaborative routines and the efficiency of sharing tacit knowledge [14], as mutual trust and close interaction among inventors take significant time to develop [33]. Second, changing relationships from weak to strong is costly [34]. Consequently, a volatile team may increase the team’s costs of forming and maintaining new collaboration, thus decreasing the team’s technological performance. Third, technological invention practices are interrupted and undermined by relational risks and opportunism if a high rate of membership turnover disrupts the continuity of team collaboration and cohesion [14, 32]. Therefore, we propose the following hypotheses:

H2a: Membership turnover exerts an inverted U-shaped effect on the team’s invention quantity.
H2b: Membership turnover exerts an inverted U-shaped effect on the team’s invention quality.

### The mediating effect of membership turnover

The scientists within a team have positive effects on membership turnover. Team membership turnover can be divided into two dimensions: adding of new members and departing of old members [15, 32]. Scientists within a team affect membership turnover by facilitating the addition of new members and the departure of old members.

The probability of an individual dropping out of the team increases with the number of scientists within the team. Scientific research and technological invention are completely different innovation practices, following different research norms and institutional logic [35]. Scientific research pursues the exploratory nature of technology whereas technological innovation pursues the prospect of commercializing technologies [25]. Consequently, forming a highly aligned emotional contract between scientists and pure inventors in an invention team is challenging, which raises the risk of dissolution of the collaborative relationship between team members. Moreover, the nature of science and technology systems is the opposite [36]. It is challenging for scientists and inventors to develop common standards of action and knowledge-based trust because of their different systems of knowledge production. Consequently, conflict frequently arises between pure inventors and scientists regarding the research focus, timing, and publication strategies during collaborative efforts, escalating the likelihood of team members leaving.

Scientists within an invention team act as catalysts for recruiting new members. Firstly, they influence the departure of existing members, creating opportunities for new members to join the team [32]. Secondly, they increase the team’s attractiveness to outside talent and the interest of outside talent in working with the team. Specifically, an invention team can rely on scientific knowledge to promote innovation diffusion such that the team’s invention results are widely disseminated to external innovators [22], attracting more innovative talents to participate in the team. Thirdly, the exploratory inventions scientists bring usually involve substantial technological knowledge, motivating the team to explore new fields and carry out new projects, requiring the team to seek innovative talent continuously.

As mentioned in Hypotheses 2a and 2b, membership turnover has an inverted U-shaped relationship with the invention quantity and quality. Thus, membership turnover plays a mediating role in how scientists within a team affect technological performance. Scientists within a team reflect on the team’s investment in scientific research and ability to apply scientific knowledge to inventions. The configuration of team members must be optimized by adding new members and removing old members to utilize scientific achievements more effectively and contribute to technological inventions. Overall, scientists within a team trigger a transmission mechanism that affects the team’s technological performance through the mediating effect of membership turnover. Hence, we propose,

H3a: Membership turnover mediates the inverted U-shaped relationship between the scientists within an invention team and invention quantity.
H3b: Membership turnover mediates the inverted U-shaped relationships between the scientists within an invention team and invention quality.

### The moderating effect of team internationalization

In the context of globalization, as technological complexity grows rapidly, innovation managers have realized the importance of internationalization as a determinant of innovation performance [19, 37]. The degree of team internationalization describes the diversity of actors in an invention team from different countries. Team internationalization challenges team management, resources, and structures [38, 39].

First, greater team internationalization indicates that invention teams involve an increasing number of members with different cultures and social values [40], and team managers are likely not able to pursue the balance between different cultures. This can complicate the communication process within the team and the relationships between old members and new arrivals. Consequently, increasing the degree of internationalization can further diminish collaboration quality in a moderately dynamic team, thus taxing the team’s collaboration management.

Second, although team internationalization may provide a team with a greater variety of knowledge and resources [41], it imposes more barriers to membership turnover. For example, having more international members familiarizes teams with heterogeneous resources and directly internalizes some of these resources. However, as internal resources are rich and heterogeneous within a given team, the relative heterogeneity of resources between a given team and others decreases. In other words, the knowledge produced by other teams can become less peculiar to a given invention team. Hence, an invention team with higher internationalization will become more accessible to the redundant pool of knowledge and resources from other teams and lose the distinct advantage of drawing on membership turnover to access diverse knowledge.

Third, according to the literature, membership turnover can introduce pronounced changes in the actors and collaboration structure within an invention team [26, 32]. According to this logic, for a great international team, an increase in membership turnover may drive the loss of valuable members and undermine the team’s international structure. As a result, the technological performance associated with the moderate membership turnover in a large international team may decline.

In sum, this study posits that team internationalization weakens the inverted U-shaped effect of membership turnover on technological performance, meaning that a highly international team benefits less from a moderate rate of membership turnover. Hence, we propose:

H4a: Team internationalization moderates the inverted U-shaped relationship between membership turnover and invention quantity and weakens this relationship.

H4b: Team internationalization moderates the inverted U-shaped relationship between membership turnover and invention quality and weakens this relationship.

### The moderating effect of team coreness

Team coreness refers to the extent to which a team contains core inventors. Core inventors were defined as those who maintained extensive and strong partnerships with both core and peripheral inventors in the inventor’s collaboration network [20]. A core inventor is deeply embedded in the co-inventor network through multiple connections with many inventors. Thus, core inventors can ensure that they have redundant channels to access the knowledge base of the other inventors, thereby opening wider conduits for searching for knowledge and resources [32]. In turn, the possibility of any given member within an invention team possessing crucial and unique knowledge and resources may decrease because of the team’s increased number of core inventors. In other words, the more core inventors there are in an invention team, the fewer participants will have a unique advantage in expertise and resources. This leads to the conclusion that team coreness makes the invention team less vulnerable to losing crucial members resulting from membership turnover.

Core inventors often possess high levels of social capital and resources, they can access to multiple regions of the co-inventor network [42]. We identify the invention team as a highly dense region in the co-inventor network. An invention team with high coreness is more likely to receive broader and faster access to the knowledge base from other teams. Furthermore, core inventors can express and share innovation ideas more efficiently with teams or individuals [43], which enables them to bridge the communication and collaboration gap between their teams and other teams or individuals in the co-inventor network. Meanwhile, these accumulated collaborative experiences and trust not only attract more inventors from other teams to the invention team but also help the invention team more effectively absorb and integrate the fresh knowledge brought by new members [2, 33].

In summary, we anticipate that the extent to which a team’s technological performance can benefit from its membership turnover depends on team coreness, which measures the degree of occupying core inventors. Specifically, we propose the following hypotheses:

H5a: Team coreness moderates the inverted U-shaped relationship between membership turnover and invention quantity and strengthens this relationship.

H5b: Team coreness moderates the inverted U-shaped relationship between membership turnover and invention quality and strengthens this relationship.

### The integrative moderated mediation model

Combining Hypotheses 3a, 3b, 4a, and 4b, we argue for an integrative moderated mediation model. The model shows that team internationalization moderates scientists’ indirect effect on technological performance. When an invention team is highly internationalized, the indirect effect of scientists on technological performance through membership turnover decreases. Similarly, according to Hypotheses 3a, 3b, 5a, and 5b, we argue that team coreness moderates scientists’ indirect effect on technological performance. When an invention team occupies more core inventors, the indirect effect of scientists on technological performance through membership turnover is amplified. Therefore, we propose the following hypotheses:

H6a: Team internationalization weakens the indirect effect of scientists in an invention team on invention quantity through membership turnover.

H6b: Team internationalization weakens the indirect effect of scientists in an invention team on invention quality through membership turnover.
H6c: Team coreness strengthens the indirect effect of scientists in an invention team on invention quantity through membership turnover.
H6d: Team coreness strengthens the inverted U-shaped effect of scientists in an invention team on invention quality through membership turnover.

**Fig 2:**
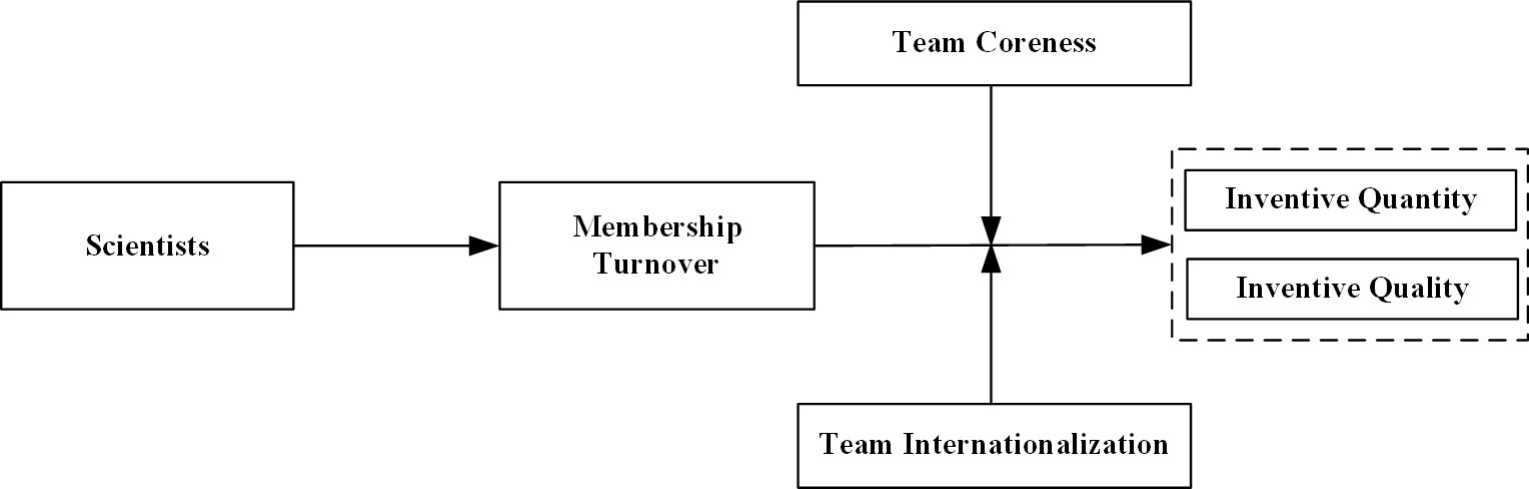
Research model.

Based on the above theoretical analyses and hypotheses, Fig 2 illustrates the underlying framework of this study.

## Materials and methods

### Data

To test our hypotheses accurately, we chose unbalanced panel data of Huawei and Intel inventors from 2000–2022. Huawei and Intel are representative corporations in an innovation-driven industry, with extensive achievements in science and technology innovation. Therefore, a thorough understanding of science–technology interactions is essential for Huawei and Intel to create and apply knowledge. In this investigation, choosing a proper sample boundary is particularly important. We emphasized co-invention relationships at the team level in two different companies, as these are more relevant and measurable to scientists within the team, membership turnover, and two dimensions of technological performance.

The study’s primary variables were computed based on scientific articles and patent data from Huawei and Intel. We extracted 31472 patents for Huawei and 49512 patents for Intel from the widely used USPTO granted between 2000 and 2022. Additionally, we collected 10305 scientific articles from Huawei and 20078 articles from Intel from the WOS database. We observed a team’s membership turnover by constructing five-year windows (i.e., 2002–2006, 2003–2007, ……, 2018–2022), resulting in 17 snapshots of collaboration networks. The regression models used a time lag between the independent and dependent variables. For instance, if the independent variables are measured during 2002–2006, then the dependent variables are measured during 2005–2006.

We use the Louvain algorithm in the Pajek software package to detect invention teams, which extracts the team structure by calculating and comparing the value of modularity as $\frac{1}{2m}\sum_{ij}\left(A_{ij}-\frac{d_{i}*d_{j}}{2m}\right)\varphi (i,j)$ [44]. Where A_ij_ is the number of inventor i’s relations involved in the linkage with inventor j, d_i_ is the number of inventor i’s collaborations, d_j_ is the number of inventor j’s collaborations, m is the sum of all the weights of the ties in an inventor network, and φ(i,j) is 1 if inventors i and j belong to the same team and 0 otherwise. A modularity value greater than 0.3 typically indicates a strong team structure in the co-inventor network and a clear boundary between invention teams [32]. We identified invention teams by examining the maximum value of network modularity, which was greater than 0.3.

**Fig 3:**
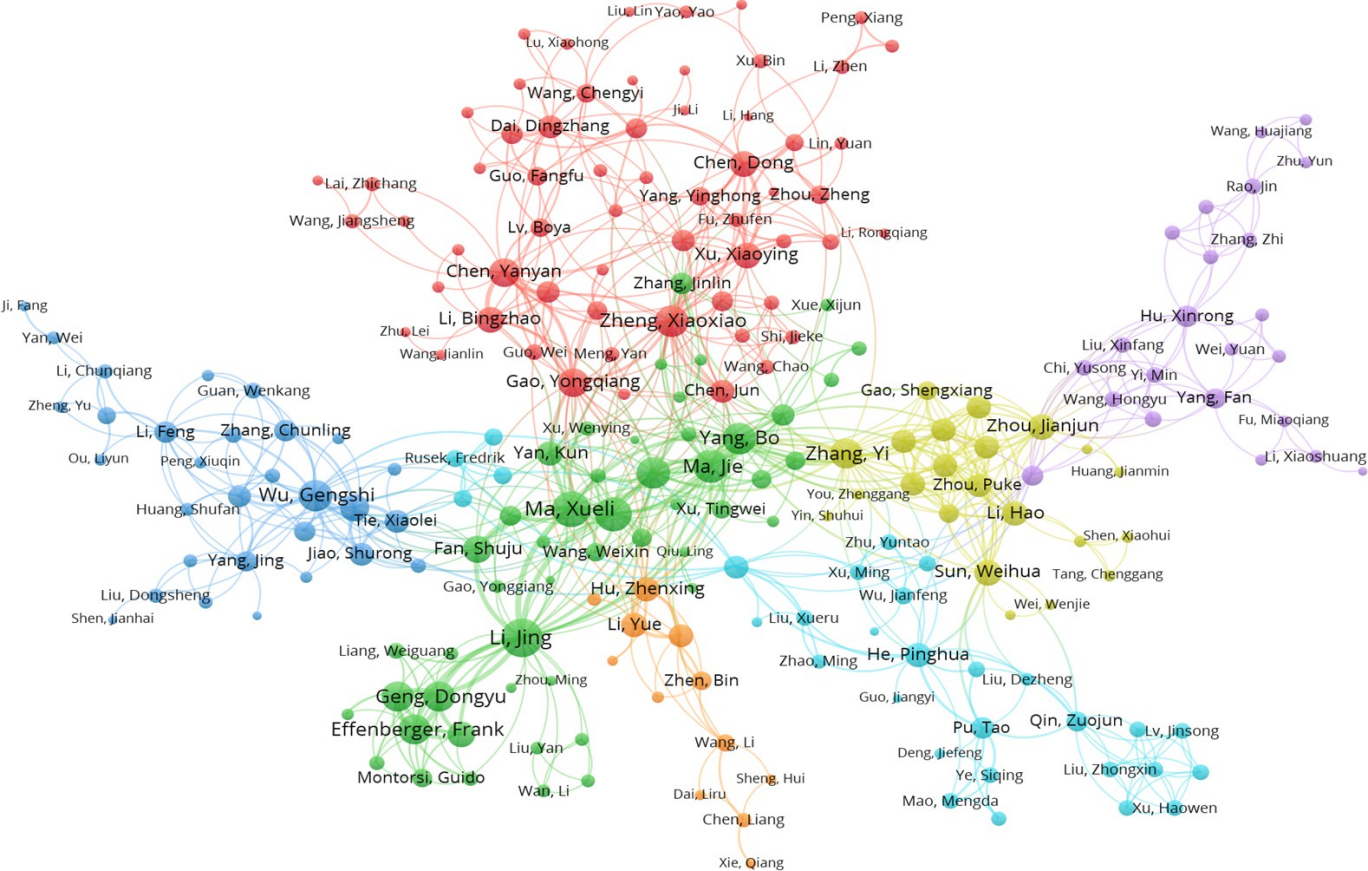
Main invention teams from Huawei Corp, 2010–2014.

**Fig 4:**
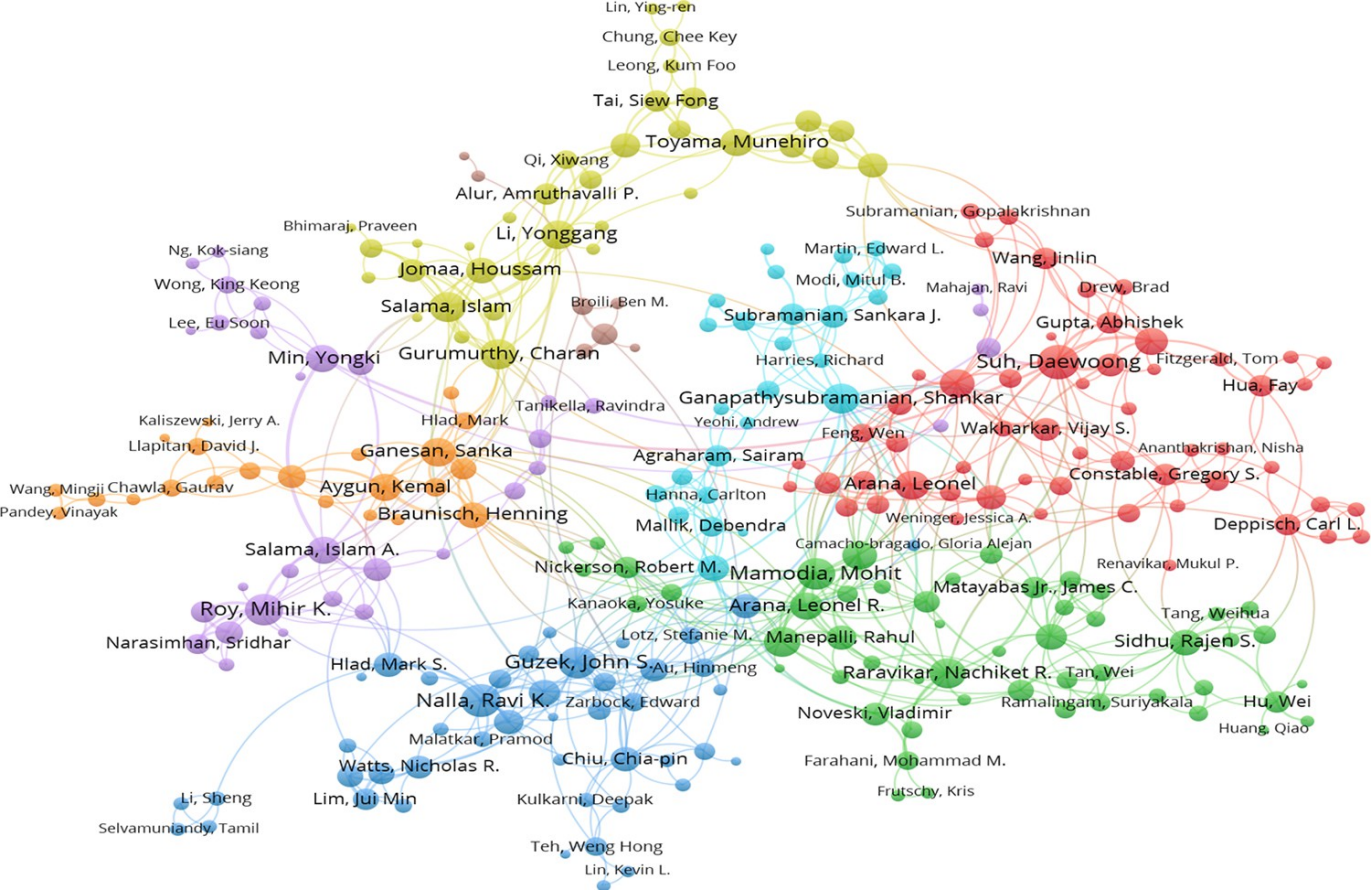
Main invention team from Intel Corp, 2010–2014.

To trace the changes in the invention team over time, we matched them across consecutive periods by using the overlap between two teams as $\frac{M_{i,t-1}⋂M_{j,t}}{M_{i,t-1}⋃M_{j,t}}$. Here, M_i,t−1_ refers to the set of members in team i in observation period t-1, M_j,t_ refers to the set of members in team j in observation period t. According to this criterion, we defined teams i and j as one team if the overlap between them was at least 30% and there was no other greater match. In Huawei Corporation, we extracted 2261 distinct teams over 17 continuous periods, and the average lifespan of invention teams was 2.98 years. In Intel Corporation, we identified 3665 teams; the team’s average duration can last 3.55 years. Figs 3 and 4 display the network structure of Huawei’s and Intel’s main invention teams between 2010 and 2014, respectively.

### Variables

#### Dependent variables

***Technological performance*. **We measure a team’s technological performance in two ways: invention quantity and quality. First, we calculated invention quantity based on the counts of a team’s successful patent applications in period t. Second, previous research demonstrates that forward citations are particularly relevant for measuring the influence of the focal patent on subsequent inventions [4, 31, 45, 46]. Thus, we calculated invention quality using the average citation of patents filed by a team.

#### Independent variables

***Scientists within an invention team*.** This indicates the percentage of scientists in the team. First, we counted the number of scientists in a team who published at least one scientific article in each period. Second, we measured the independent variable as the extent to which the team was comprised of scientists. We calculated the number of scientists in an invention team in each period as Eq (1). *PS*_*i*,*t*_ refers to the percentage of scientists within team i in period t, *T*_*i*,*t*_ refers to the size of team *i* in period t, and *S*_*i*,*t*_ is the number of scientists within team i in period t. The observation period t is five-year rolling windows, such as 2002–2006, 2003–2007, …, 2018–2022.


PSi,t=Si,tTi,t (1)

#### Mediating variables

***Membership turnover*.** To Hypotheses 2a and 2b, we defined membership turnover as the extent of changes for members within an invention team from year t-1 to t [14, 15, 32]. As shown in Eq (2), we calculated a team’s membership turnover as the inverse of team overlap across two continuous periods.


$${\mathrm{Membership}\;\mathrm{Turnover}}_{i,t}=1‐\frac{\left(M_{i,t‐1}\cap M_{i,t}\right)}{\left(M_{i,t‐1}\cup M_{i,t}\right)}$$ (2)

#### Moderating variables

***Team internationalization*.** To calculate this variable, we compute the number of countries in which each member of the invention team resides [2].

***Team coreness*:** Following Borgatti and Everett (1999) to measure team coreness, we first computed each inventor’s K core in a co-inventor network available in the Pajek software package [20]. Second, each inventor’s core / peripheral position was identified based on the average K core. Inventors are core members if their K core value exceeds the average value. Finally, we capture to what extent each team in a firm has core inventors. As indicated in Eq (3), *Team coreness*_*i*,*t*_ refers to the coreness of team *i* in period t, C_*i*,t_ refers to the number of core inventors involved in team *i*, and *T*_*i*,*t*_ is the size of team *i*.


TeamCorenessi,t=Ci,tTi,t (3)

### Control variables

Several control variables are employed to ensure robust results, and we control for a range of other inventor-level and team-level determinants of technological performance.

We specified team size as the total number of inventors who were team members in period t. Prior studies have argued that subject categories from WOS reflect the science fields involved in scientific publications [47]. We defined scientific diversity as the extent to which a team’s articles were distributed over different subject categories. Specifically, this was measured by calculating the number of subject categories involved in the team’s articles. International patent classification (IPC) is regarded as a good proxy of technological elements [31, 46]. We controlled technological diversity as the number of four-digit IPC involved in the team’s patents. A team’s prior inventions may have affected the current inventions. Hence, we controlled for the team’s invention quantity acquired in five years before the observation period. Considering the possible effects of cognitive capital on membership turnover and invention outcomes, the study computed an inventor’s cognitive capital by utilizing research tenure, the time between the current year and the year the inventor first applies for a patent [26]. To address the team’s cognitive capital, we utilized the average research tenure of the team’s inventors.

### Model specification

The dependent variable, technological performance, takes non-negative integer values and is overdispersed. Considering this, a Negative Binomial model with conditional team-level fixed effects was more appropriate for our unbalanced panel data [3, 4]. Furthermore, nonparametric bootstrapping technologies can adjust for both heteroscedasticity and autocorrelation issues and provide error estimates with minimal bias by resampling with replacement of the original sample [26]. We regressed our negative binomial models with the bootstrapping method and computed the variance of all estimates for 2000 replications.

In addition, the mediating variable, membership turnover, is a category of limited dependent variables (LDV) with values from 0 to 1, thus utilizing the Tobit model to check the first stage of the moderated mediation model [2]. Regression analyses were conducted using Stata 17.0.

## Results

Tables 1 and 2 present descriptive statistics and bivariate correlations for Huawei and Intel, respectively. The Variance Inflation Factor (VIF) for all variables was below the common threshold of 10.0, indicating the absence of problematic multicollinearity. Our estimation was further supported by condition indices within the recommended range, confirming that multicollinearity posed no serious threat.

**Table 1:** Descriptive statistics and bivariate correlations from Huawei Corp.

Variables	(1)	(2)	(3)	(4)	(5)	(6)	(7)	(8)	(9)	(10)	(11)
(1) Invention quantity	1										
(2) Invention quality	0.010	1									
(3) Scientists	0.182*	-0.058*	1								
(4) Membership turnover	0.120*	0.015	0.147*	1							
(5) Team internationalization	0.672*	-0.016	0.230*	0.161*	1						
(6) Team coreness	0.277*	0.124*	0.060*	0.053*	0.313*	1					
(7) Team size	0.760*	-0.001	0.193*	0.174*	0.832*	0.373*	1				
(8) Scientific diversity	0.653*	-0.050*	0.483*	0.217*	0.747*	0.322*	0.824*	1			
(9) Technological diversity	0.946*	-0.021	0.178*	0.110*	0.641*	0.263*	0.702*	0.624*	1		
(10) Prior invention	0.842*	-0.037*	0.149*	0.107*	0.597*	0.227*	0.663*	0.593*	0.860*	1	
(11) Cognitive capital	0.081*	-0.202*	0.022	-0.197*	0.071*	0.027	0.061*	0.073*	0.091*	0.140*	1
VIF			1.590	1.110	3.440	1.170	5.930	4.940	4.440	4.040	1.080
Mean	13.555	6.347	0.098	0.400	1.429	0.159	12.192	1.491	17.552	12.102	2.861
Sts.Dev	53.217	15.326	0.200	0.433	1.188	0.313	28.313	3.351	70.17	56.344	2.417

* p<0.01

**Table 2:** Descriptive statistics and bivariate correlations from Intel Corp.

Variables	(1)	(2)	(3)	(4)	(5)	(6)	(7)	(8)	(9)	(10)	(11)
(1) Invention quantity	1										
(2) Invention quality	0.008	1									
(3) Scientists	0.109*	-0.017	1								
(4) Membership turnover	0.149*	0.071*	0.112*	1							
(5) Team internationalization	0.714*	0.027*	0.107*	0.152*	1						
(6) Team coreness	0.286*	0.127*	0.041*	0.094*	0.338*	1					
(7) Team size	0.845*	0.013	0.121*	0.168*	0.803*	0.371*	1				
(8) Scientific diversity	0.667*	-0.003	0.427*	0.174*	0.647*	0.322*	0.777*	1			
(9) Technological diversity	0.553*	0.002	0.093*	0.133*	0.557*	0.226*	0.663*	0.605*	1		
(10) Prior invention	0.703*	-0.030*	0.094*	0.104*	0.542*	0.216*	0.674*	0.567*	0.462*	1	
(11) Cognitive capital	-0.012	-0.181*	-0.010	-0.061*	-0.051*	-0.065*	0.002	-0.018	-0.019	0.049*	1
VIF			1.450	1.040	2.870	1.180	5.530	3.770	1.880	1.860	1.020
Mean	7.570	6.020	0.140	0.330	1.590	0.140	12.190	3.210	5.070	14.150	8.380
Sts.Dev	28.840	20.240	0.230	0.400	1.480	0.300	30.240	7.010	12.910	70.910	5.570

* p<0.01

Table 3 presents the results of Huawei’s regression analyses, while Table 4 illustrates the results for Intel. Both tables report outcomes from panel Tobit models (models 1–2) and negative binomial model with team-level fixed effects and bootstrap standard errors (models 3–14). Models 8 and 14 represent the fully specified regressions incorporating all predicted effects.

**Table 3:** Panel Tobit regression model and Negative Binomial regression models with fixed effects and bootstrap standard errors, Huawei Corp (6737).

Variables	Tobit regression		Negative Binomial regression											
	Membership turnover		Invention quantity						Invention quality					
	Model 1	Model 2	Model3	Model 4	Model 5	Model 6	Model 7	Model8	Model9	Model10	Model11	Model12	Model13	Model14
Team size	-0.0005	0.001	0.012***	0.013***	0.011***	0.011***	0.009***	0.01***	0.004***	0.005***	0.002**	0.002***	0.001	0.001
	(0.0003)	(0.0003)	(0.002)	(0.002)	(0.002)	(0.002)	(0.002)	(0.002)	(0.001)	(0.001)	(0.001)	(0.001)	(0.001)	(0.001)
Scientific diversity	0.038***	0.023***	0.076***	0.057***	0.041**	0.048***	0.040***	0.045***	0.083***	0.055***	0.037***	0.036***	0.035***	0.034***
	(0.003)	(0.004)	(0.013)	(0.018)	(0.017)	(0.014)	(0.015)	(0.012)	(0.006)	(0.009)	(0.006)	(0.006)	(0.006)	(0.007)
Technological diversity	0.0004***	-0.001***	0.003***	0.003***	0.003***	0.003***	0.002***	0.002***	0.001	0.001**	0.001***	0.001***	0.0005***	0.001***
	(0.0001)	(0.0002)	(0.0003)	(0.0004)	(0.0004)	(0.0003)	(0.0003)	(0.0003)	(0.0003)	(0.0002)	(0.0002)	(0.0002)	(0.0002)	(0.0002)
Prior invention	0.0005***	0.001***	-0.002***	-0.001***	-0.001**	-0.001***	-0.001*	-0.001***	-0.00004	0.0001	0.0003	0.0003	0.0005*	0.001
	(0.0002)	(0.0002)	(0.001)	(0.001)	(0.001)	(0.0004)	(0.0004)	(0.0003)	(0.0004)	(0.0004)	(0.0003)	(0.0003)	(0.0003)	(0.0003)
Cognitive capital	-0.072***	-0.072***	-0.116***	-0.115***	-0.1***	-0.093***	-0.091***	-0.084***	-0.275***	-0.275***	-0.24***	-0.241***	-0.242***	-0.243***
	(0.005)	(0.005)	(0.027)	(0.028)	(0.023)	(0.023)	(0.024)	(0.025)	(0.04)	(0.038)	(0.034)	(0.035)	(0.035)	(0.035)
Scientists		0.321***		2.604***	2.154***	2.044***	1.994***	1.886***		1.226***	0.804***	0.714***	0.729***	0.637**
		(0.050)		(0.232)	(0.236)	(0.221)	(0.251)	(0.26)		(0.322)	(0.272)	(0.261)	(0.263)	(0.252)
Scientists squared				-3.328***	-2.818***	-2.753***	-2.651***	-2.595***		-1.118**	-0.620*	-0.539*	-0.508	-0.425
				(0.252)	(0.261)	(0.262)	(0.278)	(0.301)		(0.350)	(0.321)	(0.313)	(0.31)	(0.300)
Membership turnover					5.071***	6.807***	3.783***	5.606***			3.570***	4.878***	3.213***	4.379***
					(0.278)	(0.363)	(0.342)	(0.422)			(0.233)	(0.447)	(0.298)	(0.481)
Membership turnover squared					-4.460***	-5.872***	-3.175***	-4.738***			-3.081***	-4.219***	-2.755***	-3.773***
					(0.319)	(0.369)	(0.374)	(0.428)			(0.217)	(0.369)	(0.294)	(0.419)
Team internationalization						0.367***		0.388***				0.291***		0.275***
						(0.068)		(0.066)				(0.074)		(0.074)
Membership turnover × Team						-1.318***		-1.439***				-1.117***		-1.033 ***
internationalization						(0.203)		(0.167)				(0.262)		(0.256)
Membership turnover squared						1.056***		1.210***				0.960***		0.891***
× Team internationalization						(0.196)		(0.147)				(0.21)		(0.205)
Team coreness							0.203***	0.001					0.156	0.091
							(0.077)	(0.068)					(0.096)	(0.099)
Membership turnover ×							4.461***	5.430***					0.997	1.302**
Team coreness							(0.517)	(0.607)					(0.648)	(0.665)
Membership turnover squared							-4.122***	-4.982***					-0.754	-1.023*
×Team coreness							(0.51)	(0.547)					(0.567)	(0.582)
Constant			-1.018***	-1.055***	-1.412***	-1.84***	-1.365***	-1.782***	-0.445***	-0.485***	-0.859***	-1.178***	-0.864***	-1.156 ***
			(0.110)	(0.121)	(0.141)	(0.175)	(0.138)	(0.177)	(0.041)	(0.047)	(0.071)	(0.134)	(0.065)	(0.131)
Wald chi2			885.2	1681.2	5779.7	7066.0	7015.3	11162.3	1002.4	1495.1	2059.2	2626.1	3169.5	4248.3
Log-likelihood			-14679.2	-14589.9	-14016.3	13951.1	-13779.9	-13699.4	-13453.0	-13437.0	-13222.7	-13208.5	-13198.1	-13186.0

Standard errors are in parentheses *** p<0.01, ** p<0.05, * p<0.1

**Table 4:** Panel Tobit regression model and Negative Binomial regression models with fixed effects and bootstrap standard errors, Intel Corp (13006).

Variables	Tobit regression		Negative Binomial regression											
	Membership turnover		Invention quantity						Invention quality					
	Model 1	Model 2	Model3	Model 4	Model 5	Model 6	Model 7	Model8	Model9	Model10	Model 11	Model 12	Model 13	Model 14
Team size	0.001**	0.001***	0.017***	0.017***	0.014***	0.013***	0.012***	0.011***	0.005***	0.005***	0.003***	0.004***	0.002*	0.003**
	(0.0003)	(0.0003)	(0.003)	(0.003)	(0.002)	(0.003)	(0.002)	(0.002)	(0.002)	(0.002)	(0.001)	(0.001)	(0.001)	(0.001)
Scientific diversity	0.010***	0.004***	0.031***	0.025***	0.017***	0.014***	0.015***	0.013***	0.032***	0.023***	0.016***	0.014***	0.013***	0.011***
	(0.001)	(0.001)	(0.003)	(0.003)	(0.003)	(0.003)	(0.002)	(0.003)	(0.002)	(0.002)	(0.002)	(0.002)	(0.002)	(0.002)
Technological diversity	0.001	0.001	-0.002	-0.001	-0.001	-0.001	-0.0005	-0.001	0.002	0.003	0.003	0.003	0.003	0.003
	(0.001)	(0.001)	(0.007)	(0.007)	(0.006)	(0.007)	(0.006)	(0.007)	(0.007)	(0.007)	(0.005)	(0.005)	(0.005)	(0.005)
Prior invention	-0.0001*	-0.0001	-0.002**	-0.002*	-0.001	-0.001	-0.001	-0.001	-0.0004*	-0.0003	0.00002	-0.00001	0.0001	0.0001
	(0.0001)	(0.0001)	(0.001)	(0.001)	(0.001)	(0.001)	(0.001)	(0.0005)	(0.0002)	(0.0002)	(0.000)	(0.000)	(0.000)	(0.0002)
Cognitive capital	-0.001***	-0.010***	-0.044***	-0.044***	-0.045***	-0.045***	-0.040***	-0.040***	-0.054***	-0.054***	-0.054***	-0.053***	-0.051***	-0.051***
	(0.002)	(0.002)	(0.010)	(0.010)	(0.009)	(0.009)	(0.008)	(0.008)	(0.007)	(0.007)	(0.006)	(0.006)	(0.006)	(0.006)
Scientists		0.241***		2.138***	2.017***	2.015***	1.738***	1.712***		1.293***	1.040***	0.996***	0.887***	0.851***
		(0.035)		(0.198)	(0.184)	(0.186)	(0.168)	(0.181)		(0.265)	(0.229)	(0.220)	(0.236)	(0.235)
Scientists squared				-2.879***	-2.714***	-2.621***	-2.297***	-2.195***		-1.56***	-1.314***	-1.221***	-1.071***	-0.996***
				(0.237)	(0.212)	(0.189)	(0.200)	(0.184)		(0.352)	(0.302)	(0.288)	(0.311)	(0.305)
Membership turnover					4.601***	6.184***	3.620***	5.073***			3.051***	4.331***	2.747***	3.907***
					(0.243)	(0.257)	(0.255)	(0.285)			(0.170)	(0.203)	(0.188)	(0.209)
Membership turnover squared					-3.984***	-5.527***	-3.025***	-4.457***			-2.304***	-3.384***	-1.976***	-2.962***
					(0.256)	(0.215)	(0.258)	(0.236)			(0.173)	(0.201)	(0.193)	(0.203)
Team internationalization						0.289***		0.279***				0.262***		0.241***
						(0.053)		(0.055)				(0.019)		(0.017)
Membership turnover × Team						-1.007***		-0.998***				-0.996***		-0.947***
internationalization						(0.173)		(0.170)				(0.076)		(0.078)
Membership turnover squared						0.948***		0.960***				0.841***		0.808***
× Team internationalization						(0.137)		(0.132)				(0.063)		(0.070)
team coreness							0.345***	0.230***					0.402***	0.317***
							(0.054)	(0.067)					(0.051)	(0.053)
Membership turnover ×							3.378***	4.227***					0.688*	1.113***
Team coreness							(0.350)	(0.395)					(0.354)	(0.369)
Membership turnover squared							-3.139***	-4.077***					-0.837*	-1.221***
× Team coreness							(0.326)	(0.370)					(0.449)	(0.458)
Constant			-1.069***	-1.117***	-1.415***	-1.724***	-1.397***	-1.667***	-1.082***	-1.127***	-1.455***	-1.787***	-1.512***	-1.806***
			(0.112)	(0.124)	(0.138)	(0.144)	(0.127)	(0.135)	(0.105)	(0.094)	(0.098)	(0.097)	(0.099)	(0.105)
Wald chi2			292.9	1607. 7	3250.1	8800.6	8728.0	10886.1	849.4	1319.3	3352.21	4149.24	4919.74	8098.7
Log-likelihood			-27056.4	-26918.2	-25959.0	25647.3	-25590.6	-25249.6	-26567.1	-26534.5	-26054.6	-26010.5	-26002.3	-25964.9

Standard errors are in parentheses *** p<0.01, ** p<0.05, * p<0.1

In models 4 and 10 (Tables 3 and 4), we tested Hypotheses 1a and 1b. These results support Hypotheses 1a and 1b, indicating that scientists within an invention team exert an inverted U-shaped effect on the invention quantity and quality. As shown in model 5, the membership turnover squared emerges as a significantly negative predictor of invention quantity (Table 3: β = -4.460, p < 0.01; Table 4: β = -3.984, p < 0.01); thus, H2a is supported. Furthermore, model 11 in Tables 3 and 4 supports H2b, indicating that a team’s membership turnover affects its invention quality in an inverted U-shaped curvilinear manner (β = -3.081, p < 0.01; β = -2.304, p < 0.01).

To test Hypotheses 3a and 3b, the study first regressed the invention quantity and quality on the scientists. Secondly, it regressed invention quantity and quality on membership turnover. Thirdly, it regressed membership turnover on the scientists. The study controlled for team size, scientific diversity, technological diversity, prior inventions, and cognitive capital in all mentioned analyses. In the first and second steps, the results demonstrated in models 4, 5, 10, and 11 (Tables 3 and 4) strongly support Hypotheses 1a, 1b, 2a, and 2b. In the third step, the results of model 2 in Tables 3 and 4 report that scientists exert a significantly positive effect on membership turnover (β = 0.321, p < 0.01; β = 0.241, p < 0.01). Moreover, in Tables 3 and 4, compared with the results of models 4 and 10, the absolute value of the regression coefficient of scientists squared in models 5 and 11 is significantly reduced, indicating that membership turnover partly mediates the inverted U-shaped relationships between scientists and the quantity and quality of inventions.

To further test mediating effects, we drew on the second-stage moderated mediation models proposed by Preacher (2007) [48] and used the bootstrapping method to test mediating effects. Table 5 reveals that, following 2000 simulations using data from Huawei and Intel, the indirect effects of scientists on invention quantity through membership turnover squared term are -0.367 and -0.329, respectively. The 90% confidential interval does not contain 0. Similarly, the indirect effects of scientists on invention quality through membership turnover squared term are -0.840 and -1.425, and the 90% confidential interval does not contain 0. Hence, our combined results support Hypotheses 3a and 3b.

**Table 5:** Indirect effect of scientists within an invention team on technological performance through membership turnover.

Predictor	Mediator	Outcome	Huawei		Intel	
			Indirect effects	90% Confidence interval	Indirect effects	90% Confidence interval
Scientists	Membership turnover^^2^	Invention quantity	-0.367	(-0.525, -0.209)	-0.329	(-0.407, -0.251)
Scientists	Membership turnover^^2^	Invention quality	-0.840	(-1.362, -0.318)	-1.425	(-2.104, -0.746)

**Fig 5:**
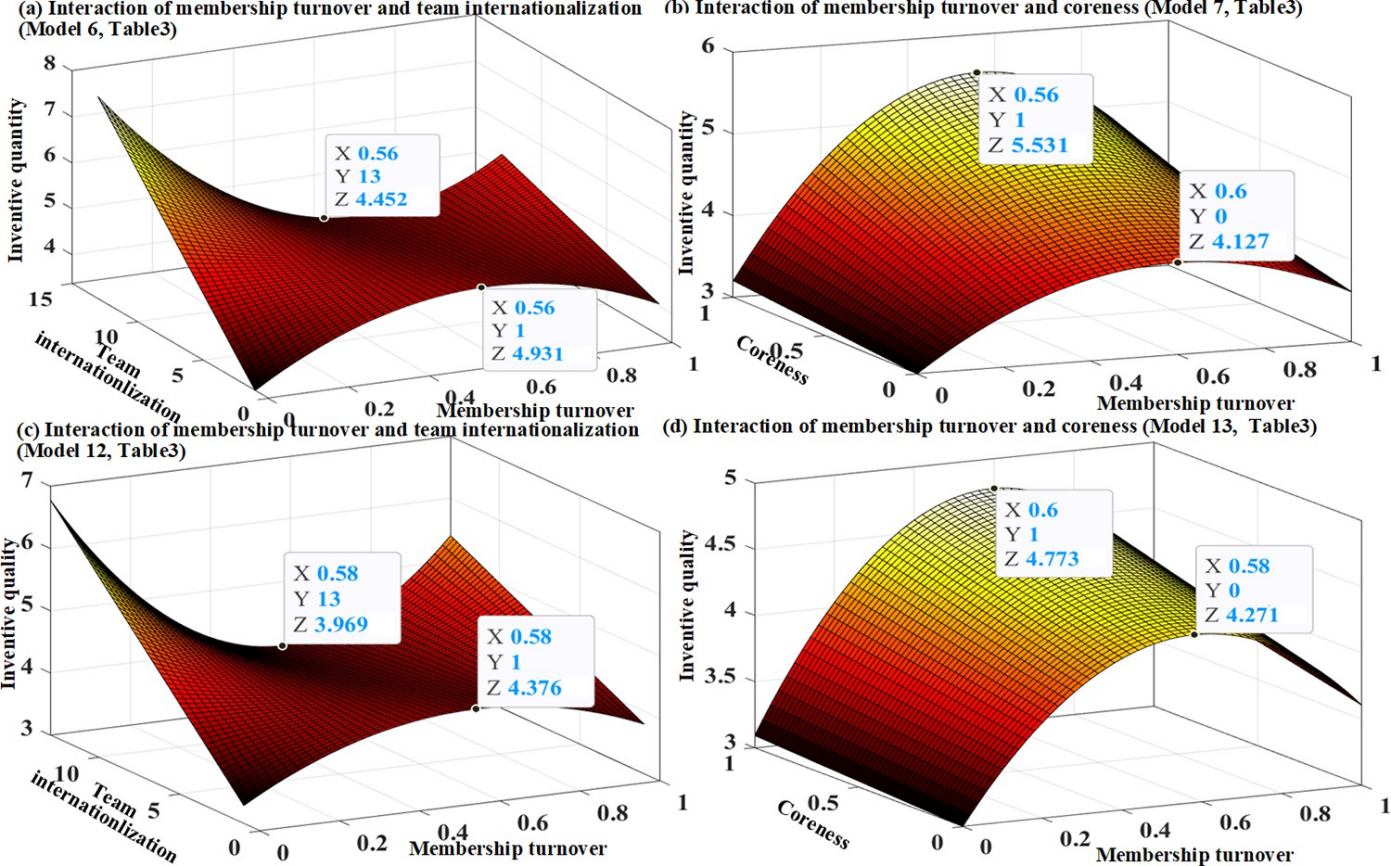
The moderation of inverted U-shaped relationships, Huawei Corp.

**Fig 6:**
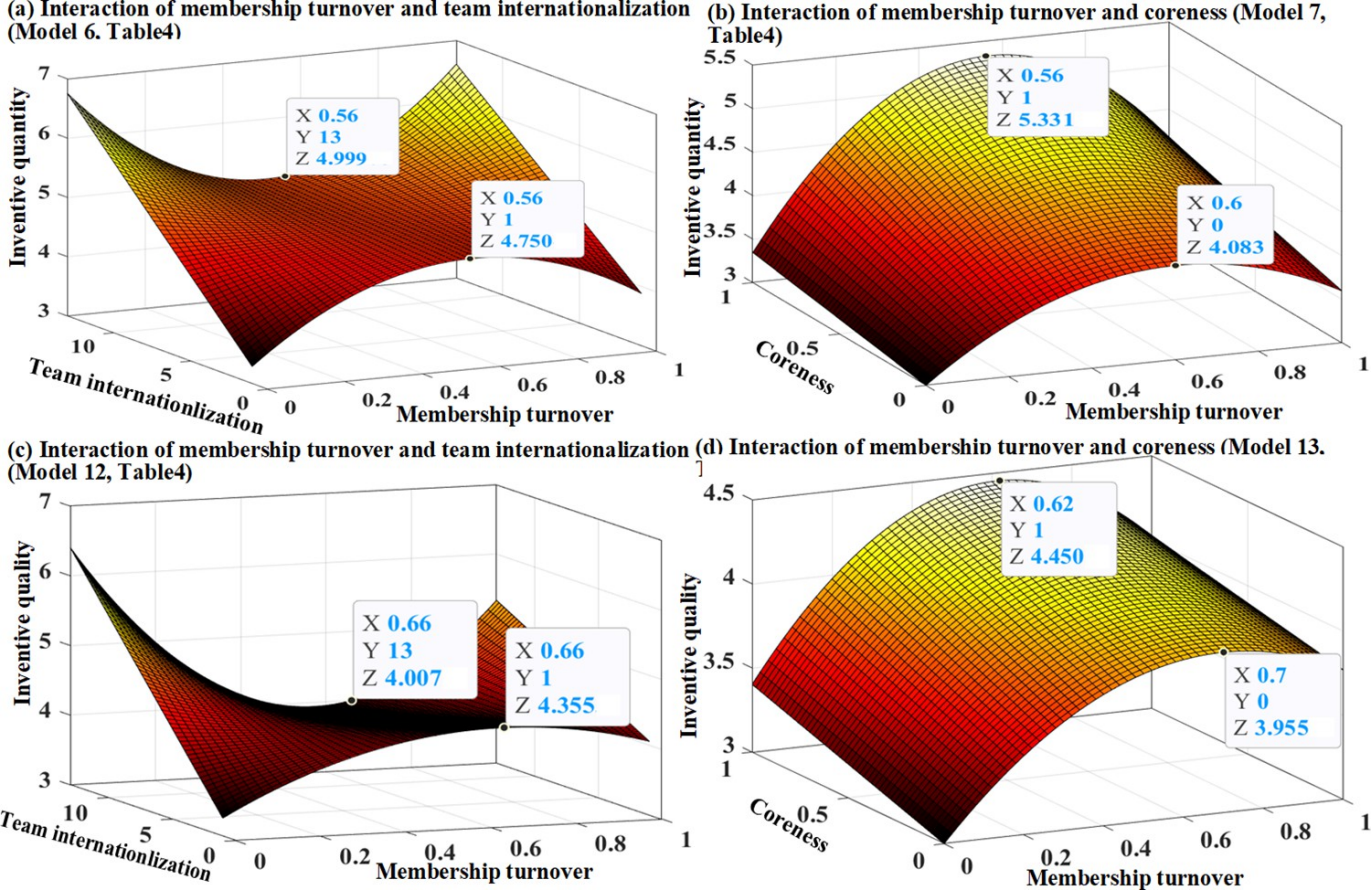
The moderation of inverted U-shaped relationships, Intel Corp.

In models 6 and 12 of Tables 3 and 4, respectively, we tested Hypotheses 4a and 4b. The results consistently support Hypotheses 4a and 4b, indicating that the inverted U-shaped effects of the membership turnover on invention quantity and quality are weakened by team internationalization. Specifically, the linear term of membership turnover shows a significantly negative interaction with team internationalization, and the coefficients of the interaction terms of membership turnover squared and team internationalization are positively significant. Therefore, the inverted U-shaped relationship between membership turnover and technological performance can be weakened by team internationalization. It can flatten the curve by weakening the positive effects of membership turnover on technological performance (see Figs 5A,5C, 6A and 6C). The results of our fully specified models 8 and 14 (Tables 3 and 4), are consistent.

In model 7 of Tables 3 and 4, Hypothesis 5a was tested. The results from Huawei and Intel consistently support Hypothesis 5a, indicating that the inverted U-shaped effect of membership turnover on invention quantity is amplified by teams coreness. Figs 5B and 6B demonstrate the study’s interesting prediction: with the increase in team coreness, a noticeable shift occurs in the inflection point in the effect of membership turnover on the invention quantity is observed, from 4.127 to 5.531 for Huawei (see Fig 5B), and from 4.083 to 5.331 for Intel (see Fig 6B). In models 13 of Tables 3 and 4, we tested Hypothesis 5b. The results support Hypothesis 5b only in the case of Intel. According to the estimates of Model 13 in Table 4, Fig 6D describes an evident nuance, where the turning point of the effect of membership turnover on invention quality changes from 3.955 to 4.450 by increasing team coreness. The prediction between membership turnover squared and team coreness was not supported by model 13 in Table 3, however, it was supported by the fully specified model 14 in Table 3. Based on the estimates of model 14, Fig 5D illustrates that the positive impact of moderate membership turnover for invention quality at Huawei is strengthened by increasing team coreness.

Hypotheses 6a-d posit that team internationalization and team coreness moderate the indirect effects of scientists on invention quantity and quality. The bootstrapping method with 2000 replications was used to test the moderated mediation effects. Table 6 reveals that the inverted U-shaped indirect effect of scientists on invention quantity through membership turnover is significant, regardless of whether team internationalization is high or low. The differences are all significant (Huawei: differences = 0.281, 90%, CI does not contain 0 (0.142, 0.419); Intel: difference = 0.284, 90% CI does not contain 0 (0.202, 0.367)). Therefore, Hypothesis H6a is supported. Similarly, Hypotheses H6b-d were also supported in the results of testing the moderated mediating effects, as indicated in Table 6.

**Table 6:** Indirect effect through membership turnover at higher and lower levels of team internationalization and team coreness.

Predictor	Mediator	Outcome	team internationalization	Huawei		Intel	
				Indirect effects	90% Confidence interval	Indirect effects	90% Confidence interval
Scientists	Membership turnover^^2^	Invention quantity	High (M+1SD)	-0.195	(-0.295, -0.096)	-0.145	(-0.196, -0.094)
			Low(M-1SD)	-0.476	(-0.680, -0.272)	-0.430	(-0.529, -0.330)
			Differences	0.281	(0.142, 0.419)	0.284	(0.202, 0.367)
Scientists	Membership turnover^^2^	Invention quality	High (M+1SD)	-0.125	(-0.205, -0.044)	-0.165	(-0.228, -0.102)
			Low(M-1SD)	-0.351	(-0.509, -0.193)	-0.292	(-0.370, -0.213)
			Differences	0.226	(0.098, 0.353)	0.127	(0.053, 0.201)
Predictor	Mediator	Outcome	team coreness	Indirect effects	90% Confidence interval	Indirect effects	90% Confidence interval
Scientists	Membership turnover^^2^	Invention quantity	High (M+1SD)	-0.465	(-0.663, -0.268)	-0.400	(-0.494, -0.305)
			Low(M-1SD)	-0.199	(-0.286, -0.111)	-0.175	(-0.222, -0.128)
			Differences	-0.267	(-0.390, -0.144)	-0.225	(-0.289, -0.161)
Scientists	Membership turnover^^2^	Invention quality	High (M+1SD)	-0.291	(-0.425, -0.156)	-0.327	(-0.412, -0.241)
			Low(M-1SD)	-0.188	(-0.283, -0.093)	-0.083	(-0.130, -0.037)
			Differences	-0.103	(-0.186, -0.019)	-0.243	(-0.326, -0.161)

The study performed robustness checks for the moderated mediation model. In the first stage, a panel OLS regression model using a bootstrapping method tested the relationship between scientists and membership turnover. For the second stage, the study explored an alternative method of calculating dependent variables (invention quantity and quality) by extending the time lag to three-year windows (2004–2006, 2005–2007, 2006–2008, ……, 2020–2022). We conducted robustness tests using the bootstrapping method again. The results in models 1–14 of Tables 7 and 8 align substantially with the original findings, meaning that our results are robust.

**Table 7:** Panel OLS regression model and Negative Binomial regression models with fixed effects and bootstrap standard errors, Huawei Corp (6737).

Variables	OLS regression		Negative Binomial regression											
	Membership turnover		Invention quantity						Invention quality					
	Model 1	Model 2	Model3	Model 4	Model 5	Model 6	Model 7	Model8	Model9	Model10	Model11	Model12	Model13	Model14
Team size	0.0003	0.001***	0.012***	0.013***	0.011***	0.011***	0.010***	0.009***	0.003***	0.004***	0.002**	0.002**	0.001	0.001
	(0.0003)	(0.0003)	(0.002)	(0.002)	(0.002)	(0.002)	(0.002)	(0.002)	(0.001)	(0.001)	(0.001)	(0.001)	(0.001)	(0.001)
Scientific diversity	0.029***	0.020***	0.074***	0.060***	0.045***	0.051***	0.044***	0.047***	0.061***	0.043***	0.035***	0.033***	0.033***	0.031***
	(0.003)	(0.002)	(0.013)	(0.016)	(0.017)	(0.013)	(0.015)	(0.010)	(0.005)	(0.006)	(0.004)	(0.004)	(0.004)	(0.004)
Technological diversity	-0.0004**	-0.0004***	0.003***	0.003***	0.003***	0.003***	0.002***	0.002***	0.001***	0.001***	0.001***	0.001***	0.001***	0.001***
	(0.0001)	(0.0001)	(0.0004)	(0.0004)	(0.0004)	(0.0003)	(0.0004)	(0.0003)	(0.0002)	(0.0001)	(0.0002)	(0.0002)	(0.0002)	(0.0001)
Prior invention	0.0003	0.0003*	-0.002***	-0.001***	-0.001**	-0.001***	-0.001*	-0.001***	-0.0002	-0.0001	0.0001	0.0001	0.0002	0.0002
	(0.0002)	(0.0002)	(0.001)	(0.001)	(0.001)	(0.0004)	(0.0004)	(0.0003)	(0.0003)	(0.0003)	(0.0003)	(0.0003)	(0.0003)	(0.0002)
Cognitive capital	-0.041***	-0.041***	-0.068***	-0.067***	-0.064***	-0.06***	-.055***	-0.050***	-0.168***	-0.168***	-0.172***	-0.173***	-0.174***	-0.175***
	(0.002)	(0.009)	(0.018)	(0.017)	(0.016)	(0.018)	(0.017)	(0.018)	(0.024)	(0.024)	(0.027)	(0.028)	(0.027)	(0.027)
Scientists		0.126***		2.353***	2.049***	1.958***	1.917***	1.829***		0.678***	0.492**	0.406**	0.434**	0.349*
		(0.030)		(0.234)	(0.240)	(0.231)	(0.268)	(0.245)		(0.236)	(0.210)	(0.191)	(0.200)	(0.186)
Scientists squared				-3.119***	-2.763***	-2.711***	-2.628***	-2.590***		-0.545**	-0.287	-0.205	-0.193	-0.113
				(0.239)	(0.245)	(0.250)	(0.275)	(0.260)		(0.272)	(0.2545)	(0.239)	(0.234)	(0.227)
Membership turnover					4.344***	5.895***	3.062***	4.729***			2.027***	3.083***	1.777***	2.674***
					(0.291)	(0.347)	(0.320)	(0.374)			(0.181)	(0.281)	(0.212)	(0.306)
Membership turnover squared					-4.028***	-5.329***	-2.761***	-4.232***			-1.994***	-2.962***	-1.774***	-2.599***
					(0.333)	(0.358)	(0.345)	(0.377)			(0.176)	(0.238)	(0.216)	(0.271)
Team internationalization						0.318***		0.341***				0.224***		0.205***
						(0.057)		(0.058)				(0.062)		(0.062)
Membership turnover × Team						-1.167***		-1.298***				-0.888***		-0.787***
internationalization						(0.179)		(0.154)				(0.204)		(0.200)
Membership turnover squared						0.956***		1.119***				0.801***		0.713***
× Team internationalization						(0.184)		(0.142)				(0.159)		(0.153)
Team coreness							0.128**	-0.028					0.176***	0.140**
							(0.06)	(0.049)					(0.066)	(0.067)
Membership turnover ×							4.706***	5.580***					0.544	0.795
Team coreness							(0.519)	(0.550)					(0.547)	(0.558)
Membership turnover squared							-4.252***	-5.079***					-0.305	-0.551
× Team coreness							(0.512)	(0.500)					(0.481)	(0.498)
Constant	0.469***	0.469***	-0.927***	-0.953***	-1.124***	-1.487***	-1.072***	-1.432***	-0.286***	-0.312***	-0.377***	-0.616***	-0.385***	-0.598***
	(0.013)	(0.023)	(0.101)	(0.106)	(0.127)	(0.158)	(0.123)	(0.150)	(0.026)	(0.070)	(0.052)	(0.094)	(0.048)	(0.093)
Wald chi2			1003.4	1942.0	4123.5	5962.9	6048.5	9371.0	1439.6	1505.2	3244.2	3333.0	3768.1	3526.8
Loglikelihood			-17004.9	-16914.2	-16446.5	-16385.3	-16162.9	-16079.6	-16046.6	-16038.8	-15967.6	-15954.5	-15943.0	-15932.0

Standard errors are in parentheses *** p<0.01, ** p<0.05, * p<0.1

**Table 8:** Panel OLS regression model and negative binomial regression models with fixed effects and bootstrap standard errors, Intel Corp (13006).

Variables	OLS regression		Negative Binomial regression											
	Membership turnover		Invention quantity						Invention quality					
	Model 1	Model 2	Model3	Model 4	Model 5	Model 6	Model 7	Model8	Model9	Model10	Model11	Model12	Model13	Model14
Team size	0.001***	0.002***	0.017***	0.017***	0.014***	0.013***	0.012***	0.011***	0.004***	0.004**	0.003**	0.003***	0.002	0.002**
	(0.0002)	(0.0004)	(0.003)	(0.003)	(0.002)	(0.003)	(0.002)	(0.002)	(0.001)	(0.001)	(0.001)	(0.001)	(0.001)	(0.001)
Scientific diversity	0.006***	0.002***	0.03***	0.025***	0.017***	0.014***	0.016***	0.013***	0.024***	0.019***	0.014***	0.012***	0.012***	0.010***
	(0.001)	(0.001)	(0.003)	(0.003)	(0.003)	(0.003)	(0.003)	(0.003)	(0.002)	(0.002)	(0.001)	(0.001)	(0.001)	(0.001)
Technological diversity	0.001*	0.001	-0.001	-0.001	-0.001	-0.001	-0.0003	-0.0004	0.002	0.002	0.002	0.002	0.003	0.003
	(0.0004)	(0.002)	(0.007)	(0.007)	(0.006)	(0.007)	(0.006)	(0.008)	(0.006)	(0.006)	(0.005)	(0.005)	(0.005)	(0.005)
Prior invention	-0.0001*	-0.0001*	-0.002*	-0.002*	-0.00118	-0.001	-0.001	-0.001	-0.0002	-0.0001	0.00003	0.00004	0.0001	0.0001
	(0.0001)	(0.000)	(0.001)	(0.001)	(0.001)	(0.001)	(0.001)	(0.0005)	(0.0002)	(0.0002)	(0.000)	(0.000)	(0.000)	(0.0002)
Cognitive capital	-0.006***	-0.011***	-0.030***	-0.030***	-0.033***	-0.033***	-0.029***	-0.029***	-0.045***	-0.045***	-0.045***	-0.045***	-0.043***	-0.043***
	(0.001)	(0.0003)	(0.008)	(0.008)	(0.008)	(0.008)	(0.007)	(0.007)	(0.005)	(0.005)	(0.004)	(0.005)	(0.004)	(0.004)
Scientists		0.130***		1.856***	1.822***	1.839***	1.575***	1.569***		0.884***	0.774***	0.734***	0.651***	0.618***
		(0.015)		(0.174)	(0.166)	(0.160)	(0.147)	(0.144)		(0.219)	(0.194)	(0.192)	(0.201)	(0.202)
Scientists squared				-2.568***	-2.492***	-2.433***	-2.119***	-2.049***		-1.113***	-0.995***	-0.909***	-0.796***	-0.725***
				(0.206)	(0.188)	(0.163)	(0.166)	(0.149)		(0.300)	(0.268)	(0.262)	(0.273)	(0.275)
Membership turnover					3.913***	5.406***	2.932***	4.333***			1.803***	2.881***	1.554***	2.519***
					(0.253)	(0.260)	(0.267)	(0.279)			(0.134)	(0.160)	(0.143)	(0.167)
Membership turnover squared					-3.587***	-5.078***	-2.644***	-4.054***			-1.504***	-2.473***	-1.247***	-2.120***
					(0.271)	(0.221)	(0.274)	(0.234)			(0.141)	(0.158)	(0.157)	(0.160)
Team internationalization						0.271***		0.266***				0.207***		0.188***
						(0.053)		(0.054)				(0.019)		(0.018)
Membership turnover × Team						-0.940***		-0.953***				-0.839***		-0.792***
internationalization						(0.175)		(0.173)				(0.089)		(0.091)
Membership turnover squared						0.907***		0.936***				0.748***		0.716***
× Team internationalization						(0.141)		(0.136)				(0.076)		(0.082)
Team coreness							0.260***	0.167***					0.339***	0.281***
							(0.044)	(0.056)					(0.038)	(0.04)
Membership turnover ×							3.690***	4.483***					0.568*	0.956***
Team coreness							(0.309)	(0.361)					(0.312)	(0.340)
Membership turnover squared							-3.318***	-4.225***					-0.632*	-1.023**
× Team coreness							(0.295)	(0.34)					(0.375)	(0.402)
Constant	0.339***	0.374***	-0.944***	-0.978***	-1.135***	-1.416***	-1.107***	-1.358***	-0.703***	-0.730***	-0.884***	-1.139***	-0.926***	-1.150***
	(0.008)	(0.021)	(0.106)	(0.114)	(0.130)	(0.136)	(0.122)	(0.121)	(0.088)	(0.079)	(0.075)	(0.082)	(0.080)	(0.079)
Wald chi2			266.2	1424.4	2647.3	6586.4	10983.3	12513.6	816.4	1133. 8	2202.5	2213.7	15779.8	13258. 3
Loglikelihood			-32185.2	-32047.6	-31294.3	-30923.1	-30844.8	-30427.9	-32176.9	-32156.2	-31997.2	-31957.0	-31945.2	-31911.3

Standard errors are in parentheses *** p<0.01, ** p<0.05, * p<0.1

A potential bidirectional relationship may exist between technological performance and membership turnover. Hence, the study employs a control function approach (CFA) to estimate the results [49], with knowledge stock and knowledge stock squared as instrumental variables. Table 9, Models 1 and 2, present the first-stage results of the CFA, with membership turnover as the dependent variable. These results validate our overall measurement approach. Both IVs demonstrate a tight connection to the team’s membership turnover, thus establishing their strength (Model 1: β = 0.105, p < 0.01 and β = -0.003, p < 0.01; Model 2: β = 0.037, p < 0.01 and β = -0.001, p < 0.01). The results are in line with our prior estimations and support our Hypotheses. Models 3–14 present the results of the second stage of the CFA, where invention quantity and quality are the dependent variables and membership turnover is the independent variable, consistent with our prior estimations in Tables 3 and 4. Thus, the CFA results further substantiate our findings.

**Table 9:** Regressions using the control function method.

Variables	First stage (OLS)		Second stage (Negative Binomial regression)											
	Membership turnover		Invention quantity						Invention quality					
	Huawei	Intel	Huawei			Intel			Huawei			Intel		
	Model 1	Model 2	Model3	Model 4	Model 5	Model 6	Model 7	Model8	Model9	Model10	Model11	Model12	Model13	Model14
Team size	-0.003***	-0.001***	0.014***	0.010***	0.010***	0.016***	0.013***	0.011***	0.005***	0.002*	0.001	0.005***	0.002***	0.002**
	(0.000)	(0.000)	(0.002)	(0.002)	(0.002)	(0.003)	(0.001)	(0.001)	(0.001)	(0.001)	(0.001)	(0.002)	(0.001)	(0.001)
Scientific diversity	-0.010***	-0.001	0.047***	0.038**	0.043***	0.025***	0.010***	0.009***	0.056***	0.030***	0.028***	0.022***	0.015***	0.011***
	(0.003)	(0.001)	0(.017)	(0.018)	(0.013)	(0.003)	(0.002)	(0.003)	(0.008)	(0.007)	(0.008)	(0.002)	(0.002)	(0.002)
Technological diversity	-0.0003*	0.0003	0.003***	0.003***	0.002***	-0.002	-0.001	-0.0004	0.001**	0.001***	0.001***	0.003	0.002	0.003
	(0.000)	(0.000)	(0.0003)	(0.0004)	(0.0003)	(0.008)	(0.003)	(0.005)	(0.0002)	(0.0002)	(0.0002)	(0.008)	(0.004)	(0.005)
Prior invention	0.0001	0.000003	-0.001***	-0.0011**	-0.001***	-0.002*	-0.0002	-0.0001	0.0002	0.00002	0.0002	-0.0004	0.0001	0.0001
	(0.000)	(0.000)	(0.0004)	(0.001)	(0.0003)	(0.001)	(0.000)	(0.000)	(0.0003)	(0.0003)	(0.0004)	(0.000)	(0.000)	(0.000)
Cognitive capital	-0.072***	-0.024***	-0.125***	-0.079***	-0.070**	-0.051***	-0.008	-0.010	-0.244***	-0.232***	-0.235***	-0.064***	-0.046***	-0.046***
	(0.002)	(0.001)	(0.023)	(0.028)	(0.029)	(0.011)	(0.008)	(0.008)	(0.033)	(0.035)	(0.034)	(0.005)	(0.006)	(0.007)
Scientists	0.194***	0.147***	2.498***	2.043***	1.794***	2.130***	1.506***	1.195***	1.118***	0.830***	0.668**	1.316***	0.953***	0.796***
	(0.030)	(0.018)	(0.210)	(0.229)	(0.244)	(0.178)	(0.134)	(0.143)	(0.281)	(0.301)	(0.280)	(0.251)	(0.215)	(0.223)
Scientists squared			-3.234***	-2.776***	-2.557***	-2.821***	-2.385***	-1.867***	-0.982***	-0.654*	-0.459	-1.590***	-1.267***	-0.970***
			(0.257)	(0.259)	(0.297)	(0.214)	(0.172)	(0.170)	(0.315)	(0.347)	(0.323)	(0.336)	(0.291)	(0.297)
Knowledge stock	0.105***	0.037***												
	(0.004)	(0.001)												
Knowledge stock squared	-0.003***	-0.001***												
	(0.000)	(0.000)												
Membership turnover				4.848***	5.346***		5.941***	6.450***		4.253***	5.082***		3.535***	4.226***
				(0.370)	(0.507)		(0.334)	(0.287)		(0.332)	(0.579)		(0.302)	(0.316)
Membership turnover squared				-3.791***	-4.079***		-2.956***	-3.868***		-3.529***	-4.279***		-2.358***	-3.006***
				(0.408)	(0.559)		(0.381)	(0.242)		(0.349)	(0.564)		(0.234)	(0.272)
Team internationalization					0.388***			0.262***			0.269***			0.235***
					(0.068)			(0.045)			(0.071)			(0.019)
Membership turnover × Team					-1.415***			-0.979***			-1.045***			-0.931***
internationalization					(0.179)			(0.127)			(0.258)			(0.079)
Membership turnover squared					1.164***			0.933***			0.911***			0.799***
× Team internationalization					(0.161)			(0.099)			(0.216)			(0.072)
Team coreness					0.004			0.258***			0.108			0.314***
					(0.069)			(0.054)			(0.100)			(0.054)
Membership turnover ×					5.395***			3.675***			1.311**			1.043***
Team coreness					(0.556)			(0.346)			(0.663)			(0.390)
Membership turnover squared					-4.988***			-3.547***			-1.036*			-1.139**
× Team coreness					(0.5138)			(0.347)			(0.5763)			(0.489)
First-stage residual			0.708***	-0.312	-0.250	0.750***	-2.440***	-2.054***	0.512***	-0.342***	-0.320***	0.957***	-0.516***	-0.342*
			(0.082)	(0.202)	(0.196)	(0.080)	(0.140)	(0.169)	(0.069)	(0.058)	(0.065)	(0.060)	(0.195)	(0.199)
First-stage residual squared			-1.837***	-0.941***	-0.787***	-1.365***	-0.049	0.087	-0.439***	0.649**	0.711***	-0.750***	0.229	0.147
			(0.388)	(0.289)	(0.268)	(0.312)	(0.147)	(0.157)	(0.160)	(0.288)	(0.274)	(0.141)	(0.143)	(0.166)
Constant	0.497***	0.431***	-0.682***	-1.424***	-1.786***	-0.839***	-2.220***	-2.320***	-0.482***	-1.090***	-1.381***	-0.922***	-1.680***	-1.945***
	(0.008)	(0.009)	(0.185)	(0.168)	(0.204)	(0.148)	(0.158)	(0.168)	(0.055)	(0.063)	(0.124)	(0.087)	(0.107)	(0.108)
Observations	6737	13006	6737	6737	6737	13006	13006	13006	6737	6737	6737	13006	13006	13006
Pseudo R2	0.190	0.092												
Log-likelihood			-14366.5	-13985.6	-13673.9	-26719.3	-25303.7	-24677.6	-13374.4	-13204.9	-13165.7	-26230.4	-26045.2	-25960.8
Wald chi2			1332.2	6012.6	8806.8	1949.3	6030.5	23927.6	1480.8	2628.0	3272.3	1544.5	5428.1	10062.9

Standard errors are in parentheses *** p<0.01, ** p<0.05, * p<0.1

## Discussion

This study represents one of the first attempts to explore the correlation between science-technology interactions, network dynamics, and technological performance. Existing studies have often delineated technological performance based on invention quantity and quality [4, 31]. Hence, this study aims to elucidate the impact of scientists within an invention team on both dimensions of technological performance. Specifically, we investigate how this relationship is mediated by membership turnover, and further explore how this mediating effect is moderated by team internationalization and coreness.

The findings confirm that scientists in an invention team have an inverted U-shaped effect on the invention quantity and quality, with a moderate number of scientists contributing positively to the team’s technological performance. Second, membership turnover is related to technological performance, and scientists within the team are associated with and influence membership turnover. Third, the study’s findings indicate that team internationalization and team coreness moderated the mediating effect of membership turnover. Specifically, team internationalization decreased the advantages of membership turnover, while the team with high coreness can benefit more from moderate rate of membership turnover.

The conclusions of this study offer several theoretical implications. Previous studies explored the direct relationship between science and technological innovation from the perspectives of scientists and scientific knowledge [2, 7, 8, 17]. This study proposes several new insights into the relationships between scientists and two dimensions of technological performance at the team level. We extend the findings in three ways.

First, we empirically hypothesize the effects of the scientists within an invention team on the quantity and quality of inventions. We explain how the quantity and quality of inventions vary under the influence of scientists at the team level.

Second, prior studies have demonstrated that network dynamics can be driven by the micro foundation of cooperation networks to account for technological performance [16, 50]. This is the first study to examine membership turnover as a mediator, thereby shedding light on the internal mechanism by which the scientists within a team influence invention quantity and quality. This study demonstrates how an invention team can integrate scientific and technological knowledge through scientist and membership turnover, which contributes to the links between knowledge-based and network dynamic theories at the team level. Given the inverted U-shaped effects of scientists on technological performance and the significant mediating effects of membership turnover in our empirical findings, invention teams should recognize the potential to achieve the highest technological performance by adjusting scientists and membership turnover at moderate levels.

Third, the basic mediation model provides an incomplete narrative, where membership turnover mediates the relationship between the scientists within the team and technological performance. This study introduces team internationalization and team coreness as moderators to explore the effects of specific team-level factors on the relationship between membership turnover and technological performance. The findings reveal that the moderating effects of two moderators may yield conflicting results, making the selection between leveraging team internationalization or team coreness challenging.

The study’s findings have several managerial implications. First, they indicate that firms can facilitate invention quantity and quality by effectively integrating scientific and technological knowledge. In detail, the study recommends managers form invention teams with a moderate number of scientists, as this is associated with a balanced membership turnover, maximizing technological performance. Moreover, the impact of membership turnover is influenced by team characteristics; for instance, a highly international team weakens the indirect effect, while high team coreness, indicative of core inventors, exacerbates it. Therefore, managers should consider membership turnover, team internationalization and team coreness. For instance, when scientists leverage membership turnover to influence technological performance, managers can enhance outcomes by introducing new external participants with a lower level of internationalization at the core of their co-inventor network.

This study presents some limitations. First, our theory and hypotheses were tested within invention teams at Huawei and Intel spanning 2000 to 2022. These findings may not be universally applicable to other organizations or periods. Future research could examine these findings using multiple datasets from various firms or industries. Additionally, our identification of invention teams in Huawei and Intel employed the approach of Louvain; however, alternative methods such as the Leiden, Girvan & Newman (GN) algorithms could be explored in future studies. Further investigations may yield valuable insights by examining team manager characteristics that affect the relationship between scientists and membership turnover. Another limitation lies in the study’s failure to distinguish between star and non-star scientists. The effect of scientists on technological performance may differ across teams owing to differences in scientific influence. Notably, star scientists positively impact innovation quality but not quantity, which is attributed to the transfer of tacit knowledge to their partnerships [3, 5]. Hence, future studies should consider the relationship between the presence of star scientists and invention research at the team level.

## Supporting information

S1 Dataset (ZIP)
